# Subcortical contributions to associative learning and cognitive control in cerebellar degeneration

**DOI:** 10.3389/fpsyg.2026.1976703

**Published:** 2026-10-26

**Authors:** Cigdem Ulasoglu-Yildiz, Emre Hari, Nur Dilara Nair, Reyhan Deniz Ari, Kardelen Yildirim, Mark A. Gluck, Tamer Demiralp, Hakan Gurvit

**Affiliations:** 1 Department of Neuroscience, Aziz Sancar Institute of Experimental Medicine, Istanbul University, Istanbul, Türkiye; 2 Hulusi Behcet Life Sciences Research Laboratory, Neuroimaging Unit, Istanbul University, Istanbul, Türkiye; 3 Department of Physiology, Istanbul Faculty of Medicine, Istanbul University, Istanbul, Türkiye; 4 Graduate School of Health Sciences, Istanbul University, Istanbul, Türkiye; 5 Department of Psychology, Faculty of Economics, Administrative and Social Sciences, Işık University, Istanbul, Türkiye; 6 Center for Molecular and Behavioral Neuroscience, Rutgers University, Newark, NJ, United States; 7 Behavioral Neurology and Movement Disorders Unit, Department of Neurology, Istanbul Faculty of Medicine, Istanbul University, Istanbul, Türkiye

**Keywords:** associative learning, cerebellum, FreeSurfer, hippocampus, inhibitory control, stimulus-response learning, striatum

## Abstract

Beyond motor control, the cerebellum has increasingly been implicated in cognitive functions, including inhibitory control, whereas its contribution to feedback-based associative learning remains less well established. This study investigated the structural correlates of inhibitory control and feedback-based learning of stimulus-response (S-R) associations in cerebellar degeneration, focusing on cerebellar, striatal, and hippocampal contributions. Twenty-seven patients with cerebellar degeneration and 28 age-, sex-, and education-matched healthy controls completed the Stroop test and the Concurrent Discrimination and Transfer Test (CDTT) and underwent structural magnetic resonance imaging. The CDTT comprised two phases: acquisition of S-R associations and transfer of previously acquired associations to novel stimulus combinations. Patients showed significantly reduced right caudate volume, impaired inhibitory control, and impaired feedback-based S-R acquisition, characterized by slower acquisition and increased acquisition errors. Among participants who reached the acquisition criterion, no significant between-group difference in transfer performance was detected. In patients, Stroop interference time was negatively correlated with right hippocampal volume. Acquisition error rate was negatively correlated with left and right hippocampal and right putamen volumes, whereas the number of trials required to acquire S-R associations was negatively correlated with right hippocampal volume. In healthy controls, acquisition error rate was negatively correlated with bilateral cerebellar cortex volumes. Between-group analyses showed that the association between acquisition speed and right hippocampal volume differed between groups, whereas no significant interactions between group and regional brain volumes were observed for acquisition error rate. Together, these findings indicate impaired inhibitory control and feedback-based S-R acquisition in cerebellar degeneration. The findings support a distributed systems account of cognitive dysfunction in cerebellar degeneration and highlight differential associations of cerebellar, striatal, and hippocampal volumes with feedback-based S-R learning.

## 1 Introduction

Contemporary accounts propose that the cerebellum contributes to cognition by generating predictions about the consequences of actions and events, comparing expected and actual outcomes, and updating internal models based on prediction errors (Ito, 2008). Beyond motor control, these predictive computations have been implicated in executive functions including cognitive control, performance monitoring, response inhibition, attention, and adaptive behavior through reciprocal connections with prefrontal association cortices (Ramnani, 2006; Schmahmann, 2019). Anatomical and functional studies have further shown that the cerebellum forms reciprocal polysynaptic circuits with the basal ganglia (Hoshi et al., 2005; Bostan et al., 2010; Milardi et al., 2016; Pelzer et al., 2013; Radhakrishnan et al., 2023) and cerebral cortex (Palesi et al., 2015), supporting coordinated cerebellar and striatal contributions to adaptive behavior (Stoodley and Schmahmann, 2018).

The cognitive consequences of cerebellar damage are commonly described within the framework of cerebellar cognitive affective syndrome, which includes executive dysfunction, visuospatial deficits, language disturbances, and affective dysregulation (Schmahmann and Sherman, 1998). Executive impairments, particularly reduced inhibitory control and cognitive flexibility are among the most consistently reported cognitive manifestations of cerebellar disease (Hoche et al., 2018). Executive dysfunction also appears to be one of the most consistent cognitive findings among spinocerebellar ataxias, which are characterized by progressive cerebellar degeneration (Selvadurai et al., 2024; Tenório et al., 2025).

In contrast, hippocampal-dependent explicit memory appears to be relatively preserved following cerebellar damage. Instead, reported memory difficulties may be mediated or amplified by deficits in executive control, attention, processing efficiency, working memory, or strategic retrieval (Appollonio et al., 1961; Brandt et al., 2004). However, the contribution of the cerebellum to less effortful, feedback-based associative learning, which relies largely on striatal learning and memory systems, remains less well established. A limited number of studies have reported impairments in learning sensory associations (Timmann et al., 2010), sequential (Leggio et al., 2011; Bernard and Seidler, 2013) and contextual (Ulasoglu-Yildiz and Gurvit, 2020) regularities, although findings have varied across paradigms and learning domains.

A two-phase Concurrent Discrimination and Transfer Task (CDTT) enables the assessment of both feedback-based acquisition of stimulus-response (S-R) associations and the subsequent transfer of learned associations to novel stimulus combinations. During the transfer phase, the task-relevant feature–response relationship remains unchanged despite changes in irrelevant stimulus features (Myers et al., 2002). Studies using this paradigm have shown that patients with striatal dysfunction due to Parkinson’s disease are impaired during acquisition while exhibiting preserved transfer after successful learning (Shohamy et al., 2004). This behavioral dissociation has been attributed to disruption of frontostriatal mechanisms supporting feedback-based acquisition, whereas successful transfer has been linked to additional contributions from MTL (medial temporal lobe) systems (Myers et al., 2003).

Beyond the established contribution of frontostriatal systems, evidence suggests that the cerebellum may also contribute to learning from external feedback. Patients with isolated cerebellar degeneration have shown impairments in non-motor associative learning acquired through trial-and-error feedback (Drepper et al., 1999), and more recent evidence indicates impaired incremental rewards learning in patients with cerebellar ataxia (Nicholas et al., 2024). Electrophysiological studies further demonstrate altered feedback processing in cerebellar disorders, including altered event-related potential (ERP) responses to positive and negative feedback in patients with selective cerebellar lesions (Rustemeier et al., 2016) and absent reinforcement-learning prediction error coding in feedback-related ERP components in patients with pure cerebellar degeneration (Berlijn et al., 2025). Together, these findings provide more direct evidence for cerebellar involvement in feedback-based learning and feedback processing. Our recent study (Ulasoglu-Yildiz et al., 2025) further showed that patients with Korsakoff syndrome, a disorder primarily characterized by damage to the medial diencephalon but frequently preceded by Wernicke encephalopathy, in which cerebellar pathology may also occur, exhibited slower acquisition of simple S-R associations on the CDTT while maintaining intact transfer ability. They also showed impaired acquisition of more complex associations, with poorer acquisition performance associated with greater ataxia severity. Although this association raised the possibility of a contribution from cerebellar dysfunction, cerebellar pathology could not be isolated from the broader neuropathology of Korsakoff syndrome. Together with more direct evidence described above, these findings raise the question of whether cerebellar pathology may contribute to feedback-based S-R learning. The combined evidence therefore provides a rationale for investigating whether cerebellar degeneration affects feedback-based associative learning. Indeed, whether isolated cerebellar degeneration differentially affects the acquisition and transfer of S-R associations remains unknown.

Progressive cerebellar degeneration (Paulson, 2009; Teive and Ashizawa, 2015) provides a valuable model for examining the contribution of cerebellar pathology to cognitive control and feedback-based associative learning. The present study therefore investigated inhibitory control, feedback-based associative learning, and the transfer of learned associations in patients with hereditary cerebellar ataxias including genetically confirmed autosomal recessive or autosomal dominant cerebellar ataxias and demographically matched healthy controls using the Stroop Test and CDTT. In addition, structural magnetic resonance imaging (MRI) was used to investigate associations between cognitive performance and cerebellar, striatal, and hippocampal volumes. Based on previous evidence, we hypothesized that patients with cerebellar degeneration would exhibit impaired inhibitory control and slower acquisition of S-R associations, whereas we expected them to successfully transfer the S-R associations once acquired.

## 2 Materials and methods

### 2.1 Participants

The study included 27 patients with cerebellar degeneration, and 28 healthy controls matched one-to-one on age, sex, and years of education. The patient group comprised individuals with genetically confirmed autosomal recessive or autosomal dominant cerebellar ataxias characterized by predominantly cerebellar involvement. Patients were recruited from the hereditary ataxia outpatient cohort of the Behavioral Neurology and Movement Disorders Unit, Department of Neurology, Istanbul Faculty of Medicine, Istanbul University. Eligible patients had a chronic progressive cerebellar syndrome, a family history of a similar disorder and/or parental consanguinity, and isolated diffuse cerebellar atrophy on clinical MRI. Participants were between 18 and 65 years of age, right-handed, and had completed at least primary education. Individuals with episodic, X-linked, or mitochondrial ataxias; another neurological or psychiatric disorder; a chronic systemic condition likely to affect cognition; a history of stroke, or a history of head trauma with loss of consciousness were excluded.

### 2.2 Clinical, neuropsychological, and experimental assessment

Ataxia severity was assessed using the International Cooperative Ataxia Rating Scale (ICARS, range, 0–100) (Trouillas et al., 1997), with higher scores indicating greater ataxia severity. Participants were screened for depressive symptoms using the Beck Depression Inventory (BDI; Beck et al., 1961; Hisli, 1988). Cognitive control was evaluated with the Stroop Test (Stroop, 1935), and feedback-based associative learning and transfer were assessed using the Concurrent Discrimination and Transfer Task (CDTT).

The CDTT consisted of two phases. During the acquisition phase, participants learned the correct choice for eight object pairs through trial-by-trial feedback. Objects within a pair shared either color or shape (Figure 1, left column), while the feature that distinguished them predicted rewards. After each response, the selected object moved upward, and a smiley face appeared only after a correct choice (Figure 1, middle column). Stimulus position varied across trials to prevent learning based on spatial location. Acquisition phase continued until the participant produced 16 consecutive correct responses or completed a maximum of 96 trials. The transfer phase began immediately and without warning. During this phase, the rewarded object remained unchanged while the irrelevant shared feature was replaced (Figure 1, right column). This phase continued until 16 consecutive correct responses or a maximum of 96 trials (Sinha et al., 2019). Participants who failed to reach the acquisition criterion were excluded from transfer analyses. Acquisition speed was indexed by the number of trials required to reach the acquisition criterion, with fewer trials indicating faster acquisition. Trials to criterion, acquisition errors, and transfer errors served as behavioral measures (Myers et al., 2002, 2003).

**FIGURE 1:**
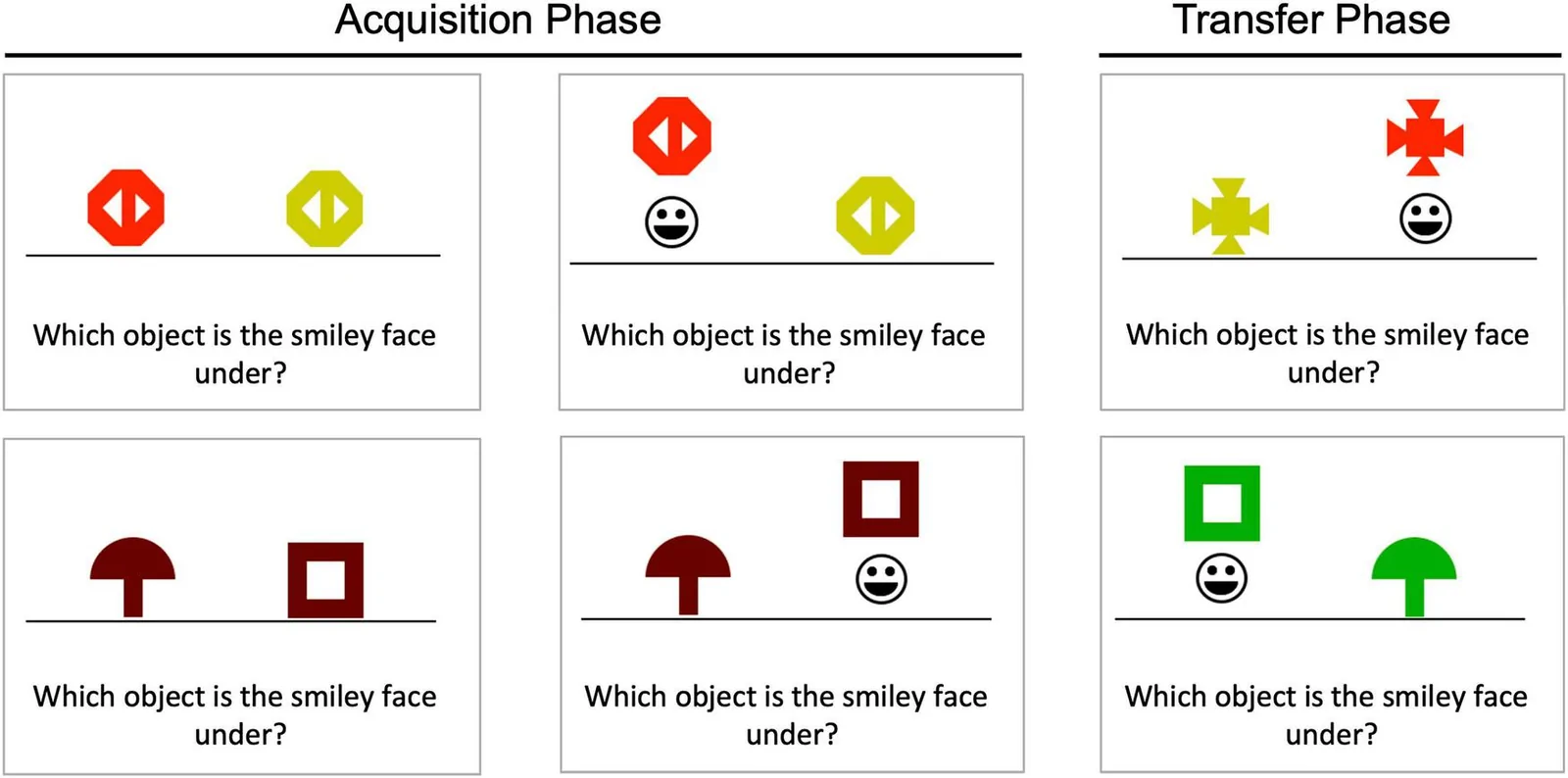
Screen events during a sample trial in the acquisition and transfer phases. On each trial, participants choose one of two objects and learn, through feedback, which object in each discrimination pair is rewarded. In the transfer phase, the objects are replaced while preserving the relevant dimension (top, color; bottom, shape) and changing the irrelevant dimension (top, shape; bottom, color).

### 2.3 MRI acquisition and data processing

High-resolution T1-weighted structural MRI images were acquired using a 3-Tesla MRI scanner (Philips, Achieva, Best, The Netherlands) equipped with a 32-channel SENSE head coil at the Hulusi Behçet Life Sciences Research Laboratory, Neuroimaging Unit, Istanbul University. A 3D TFE (Turbo Field Echo) sequence was conducted with the following parameters: repetition time (TR)/echo time (TE) = 8.4/3.9 ms, flip angle = 8°, Field of View (FOV) = 250 mm × 250 mm, matrix = 252 × 227, 180 transverse slices (without gap), 1 mm^3^ isotropic resolution.

Data were processed using the automated FreeSurfer pipeline (v 7.2.0)^1^. Standard recon-all steps were conducted to the individual data for each participant (Fischl et al., 2002) and subsequently skull-stripping and segmentation were visually inspected. Regional brain volumes were extracted separately for each hemisphere, including the left and right cerebellar cortices and the left and right caudate, putamen, and hippocampus (Figure 2).

**FIGURE 2:**
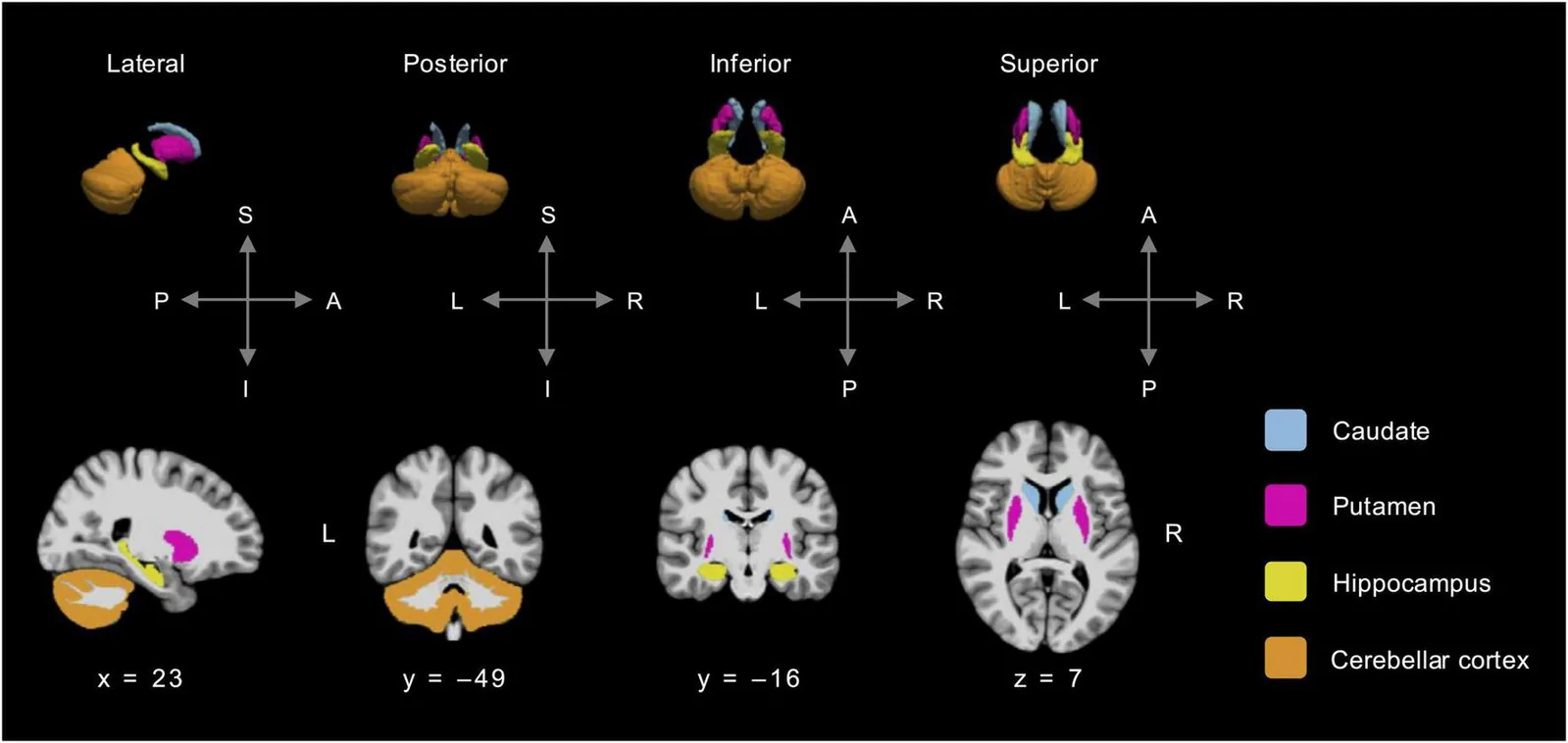
Three- and two-dimensional view of the subcortical regions and cerebellar cortex from the lateral, posterior, inferior and superior perspectives. A, anterior; I, inferior; L, left; R, right; S, superior.

### 2.4 Statistical analysis

Unless otherwise specified, statistical analyses were performed using SPSS version 24.0 (IBM Corp., Armonk, NY, USA). The normality of continuous variables was assessed using the Shapiro–Wilk test. Statistical tests were selected according to the distributional characteristics and measurement level of each variable. Accordingly, between-group comparisons were performed using the independent-samples *t*-test or Mann–Whitney U test for continuous variables, as appropriate, and the chi-square test for categorical variables. Analysis of covariance (ANCOVA) was used to examine group differences in regional brain volumes, controlling for age, sex, and estimated total intracranial volume (eTIV).

Brain–behavior correlation analyses were designed to examine whether regional brain volumes were associated with the severity of the hypothesized cognitive deficits. For the Stroop task, interference time was specified *a priori* as the primary continuous index of Stroop interference. For the CDTT, analyses focused on acquisition performance because the primary hypothesis concerned impaired acquisition of feedback-based S-R associations in the patient group, whereas transfer was hypothesized to be relatively preserved once the associations had been acquired. Transfer measures were therefore not considered indices of the hypothesized deficit and were not included in the brain–behavior correlation analyses.

Spearman’s rank correlation analyses were performed to examine associations between cognitive performance and clinical variables, including disease duration and motor symptom severity. Partial Spearman rank correlation analyses controlling for eTIV were performed in R (version 4.5.1; R Foundation for Statistical Computing, Vienna, Austria) using the *ppcor* package. First, associations between regional brain volumes and clinical variables (disease duration and motor symptom severity) were examined in patients. Subsequently, associations between regional brain volumes and Stroop interference time and between regional brain volumes and CDTT acquisition performance were examined.

To assess whether the observed brain–behavior associations were specific to individual cognitive measures rather than attributable to shared variance between behavioral measures, additional partial Spearman rank correlation analyses were performed by including the alternate behavioral measure as an additional covariate alongside eTIV. Specifically, when examining associations between regional brain volumes and Stroop interference time, CDTT acquisition performance was included as an additional covariate; conversely, when examining associations between regional brain volumes and CDTT acquisition performance, Stroop interference time was included as an additional covariate.

To explore whether the brain–behavior associations underlying the observed group-specific patterns differed between patients and healthy controls, follow-up ANCOVA models were conducted for regional brain volumes selected based on the correlation findings. Acquisition performance was entered as the dependent variable, group and sex as fixed factors, and regional brain volume, age, and eTIV as continuous covariates. The group × regional brain volume interaction was the effect of interest. All interaction analyses were controlled for age, sex, and eTIV.

False discovery rate (FDR) correction was applied separately to each family of comparisons. For the follow-up interaction analyses, the group × regional brain volume interaction for acquisition speed was tested only for the right hippocampus, and no multiple-comparison correction was applied, as this was the only region significantly associated with the number of trials to criterion in the partial correlation analyses. For acquisition error rate, FDR correction was applied across the five ROI-specific interaction tests corresponding to the significant within-group correlations. All statistical tests were two-tailed, and statistical significance was defined as *p* < 0.05, with FDR correction applied where appropriate.

To visualize the correlation findings, data distributions were illustrated using marginal density plots, and locally estimated scatterplot smoothing (LOESS) curves with 95% confidence intervals were overlaid to depict the overall trend of the data. LOESS smoothing was selected as a non-parametric approach because it does not assume a linear relationship between variables and provides a flexible representation of potential non-linear associations.

## 3 Results

The patient and control groups were demographically comparable and did not differ in depressive symptom scores (all *p*-values > 0.05). The patient group was genetically heterogeneous but shared a predominantly cerebellar neurodegenerative phenotype, which was the primary criterion for inclusion in the present study. The group showed mild-to-moderate cerebellar motor impairment (Statton et al., 2018) and had a mean disease duration of 11.6 years (Table 1).

**TABLE 1:** Demographics and clinical features of the sample.

Variable	HC (*N* = 28)	CD (*N* = 27)	Test	*P*
Sex (*n*, female/male)	12/16	12/15	χ^2^ = 0.014	0.906
Age (mean ± SD)	40.25 ± 14.23	40.37 ± 13.74	*t* = 0.032	0.975
Education (years)	10.36 ± 3.52	10 ± 4.13	*t* = 0.346	0.731
Depression (BDI)	3.68 ± 3.92	4.85 ± 3.45	*t* = 1.18	0.244
Disease duration (years)	−	11.63 ± 6.76	−	−
Ataxia severity (ICARS)	−	32.15 ± 12.30	−	−

BDI, Beck Depression Inventory; CD, patients with cerebellar degeneration; HC, healthy controls; ICARS, International Cooperative Ataxia Rating Scale; SD, standard deviation.

### 3.1 Inhibitory control, associative learning and transfer

Patients showed poorer Stroop performance, with longer interference times (*U* = 243.000, *p* = 0.036, *p*_*FDR*_ = 0.036), and more errors (*U* = 239.000, *p* = 0.008, *p*_*FDR*_ = 0.013) and spontaneous corrections (*U* = 212.500, *p* = 0.007, *p*_*FDR*_ = 0.013) (Figure 3A and Table 2). They also exhibited impaired acquisition of S-R associations, requiring more trials to reach criterion (*U* = 156.500, *p* = 1.83E-04, *p*_*FDR*_ = 3.66E-04), and making more acquisition errors (*U* = 202.000, *p* = 0.003, *p*_*FDR*_ = 0.003) than controls (Figure 3B). Eight patients and two controls who failed to reach criterion were excluded from transfer analyses. Among participants who reached the acquisition criterion, no significant between-group differences were observed in the number of transfer trials (*U* = 246.500, *p* = 0.991) and errors (*U* = 244.500, *p* = 0.953) (Figure 3C and Table 2).

**FIGURE 3:**
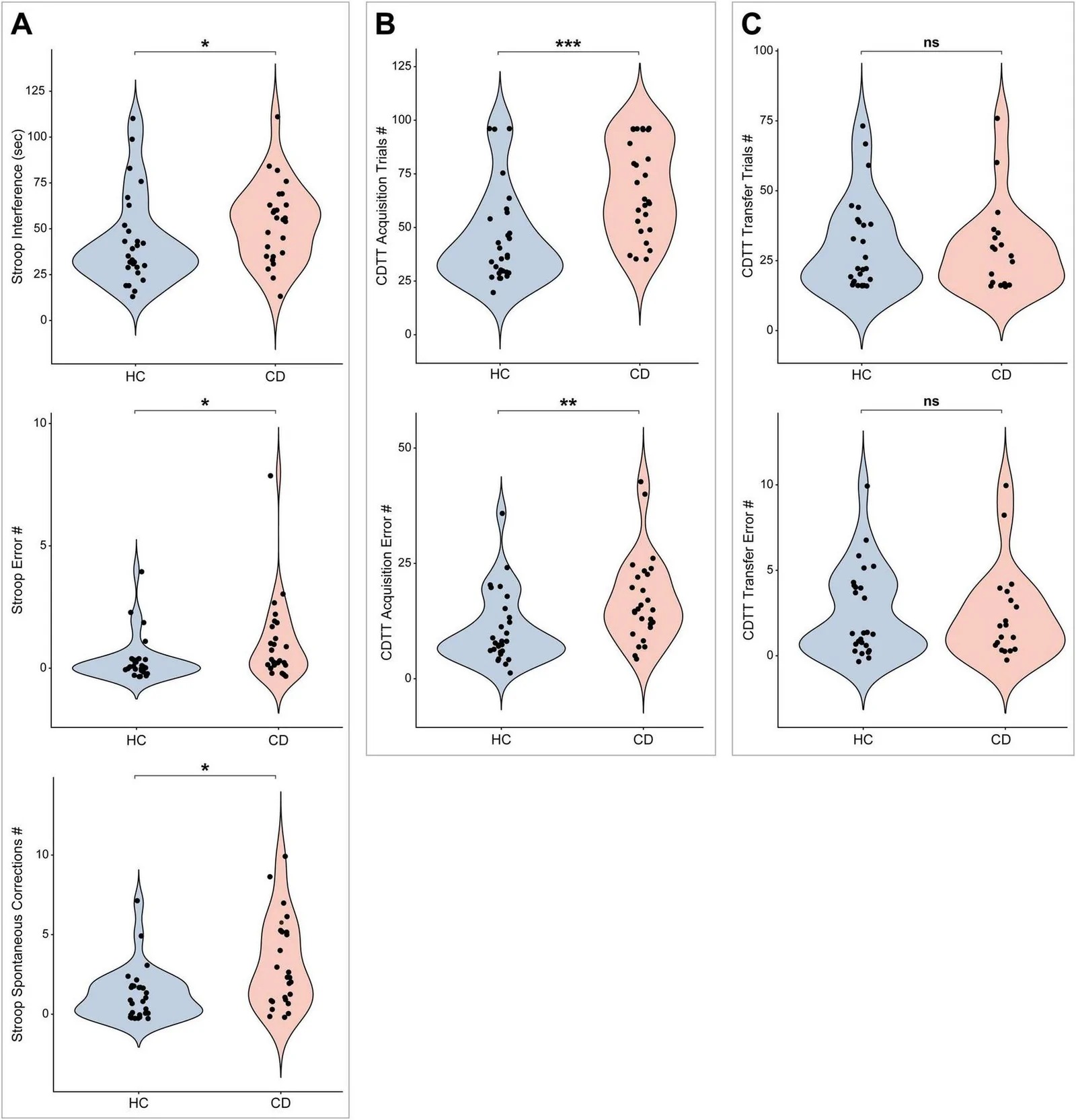
Distribution of Stroop and CDTT performance across healthy controls and patients with cerebellar degeneration. **(A)** Stroop interference time, error count, and number of spontaneous corrections; **(B)** number of trials and errors during acquisition of feedback-based S-R associations on the CDTT; **(C)** number of trials and errors during transfer of the learned S-R associations. *p_FDR_ < 0.05, **p_FDR_ < 0.01, ***p_FDR_ < 0.001; ns, not significant. # denotes numbers.

**TABLE 2:** Between-group comparisons of Stroop task and CDTT performance.

Variable	HC (*N* = 28) Mdn (min – max)	CD (*N* = 27) Mdn (min – max)	*U*	*P*	*p*_*FDR*_
Stroop interference (sec)	34 (13 – 110)	55.5 (13 – 111)	243.000	0.036	0.036*
Stroop error	0 (0 – 4)	0.5 (0 – 8)	239.000	0.008	0.013*
Stroop spontaneous correction	1 (0 – 7)	2 (0 – 10)	212.500	0.007	0.013*
CDTT acquisition trials #	36.5 (20 – 96)	63 (35 – 96)	156.500	<0.001	<0.001***
CDTT acquisition errors #	8 (1 – 36)	15 (4 – 43)	202.000	0.003	0.003**
	(*N* = 26)	(*N* = 19)			
CDTT transfer trials #	22 (16 – 73)	27 (16 – 76)	246.500	0.991	0.991
CDTT transfer errors #	1 (0 – 10)	2 (0 – 10)	244.500	0.953	0.991

CD, patients with cerebellar degeneration; HC, healthy controls; SD, standard deviation; Mdn, Median. *p* indicates uncorrected *p*-values; asterisks indicate FDR-corrected significance:

*p_FDR_ < 0.05,

**p_FDR_ < 0.01,

***p_FDR_ < 0.001. FDR-corrected significant results are shown in bold. # denotes number.

### 3.2 Structural findings

Structural MRI confirmed marked bilateral cerebellar atrophy in the patient group. Both left and right cerebellar cortices showed significant volume reduction (*p* = 3.07E-11, *p*_*FDR*_ = 3.07E-11; *p* = 1.17E-12, *p*_*FDR*_ = 2.34E-12). Among supratentorial regions, only the right caudate showed significant volume reduction after correction for multiple comparisons (*p* = 0.008, *p*_*FDR*_ = 0.048). The left caudate and left putamen also showed marginally significant volume reduction, while these differences did not survive correction (*p* = 0.027; *p*_*FDR*_ = 0.069; *p* = 0.035, *p*_*FDR*_ = 0.069). Right putamen volume and hippocampal volumes did not differ between groups (*p*_*FDR*_ > 0.05) (Table 3).

**TABLE 3:** ANCOVA results for between-group comparisons of subcortical and cerebellar volumes controlling for age, sex, and eTIV.

Region	HC *(N* = 28) Mean ± SD	CD (*N* = 27) Mean ± SD	*F*	*p*	*p*_*FDR*_	η_p_^2^
Subcortical region						
L Caudate	3468.76 ± 509.45	3179.23 ± 446.21	5.187	0.027	0.069	0.094
R Caudate	3619.69 ± 475.98	3298.67 ± 425.01	7.619	0.008	0.048*	0.132
L Putamen	4937.53 ± 786.50	4537.40 ± 642.69	4.711	0.035	0.069	0.086
R Putamen	4929.17 ± 691.96	4624.34 ± 645.59	2.968	0.091	0.137	0.056
L Hippocampus	4106.65 ± 478.35	3935.17 ± 461.90	1.622	0.209	0.250	0.031
R Hippocampus	4321.89 ± 409.20	4172.89 ± 516.26	1.204	0.278	0.278	0.024
Cerebellar cortex						
L Cerebellum	50385.17 ± 6649.79	34173.06 ± 8601.64	71.861	<0.001	<0.001***	0.590
R Cerebellum	51510.46 ± 6708.09	34066.26 ± 8080.97	88.665	<0.001	<0.001***	0.639

CD, patients with cerebellar degeneration; HC, healthy controls; L, left; R, right; SD, standard deviation. *p* indicates uncorrected *p*-values; asterisks indicate FDR-corrected significance:

*p_FDR_ < 0.05,

***p_FDR_ < 0.001. FDR-corrected significant results are shown in bold.

### 3.3 Correlational findings

Neither cognitive performance nor regional brain volumes were significantly associated with disease duration or motor symptom severity (i.e., ICARS), suggesting that the observed cognitive and structural differences could not readily be explained by either clinical variable (See Tables 4, 5).

**TABLE 4:** Spearman’s rank correlations (ρ) among Stroop performance, CDTT acquisition performance and clinical variables in CD.

Variable	Interference (sec)^1^	Trial #^2^	Error %^2^
Disease duration (years)	ρ = 0.292 *p* = 0.156	ρ = 0.171 *p* = 0.403	ρ = −0.165 *p* = 0.421
ICARS	ρ = 0.097 *p* = 0.636	ρ = 0.277 *p* = 0.161	ρ = 0.223 *p* = 0.265

1. Stroop test; 2. CDTT acquisition phase. *p* indicates uncorrected *p*-value. # denotes number.

**TABLE 5:** Partial Spearman rank correlations (ρ) between clinical variables and subcortical and cerebellar volumes controlling for eTIV.

Variable	L CAU	R CAU	L PUT	R PUT	L HIP	R HIP	L CER	R CER
Disease duration	ρ = −0.211 *p* = 0.311	ρ = −0.043 *p* = 0.837	ρ = −0.206 *p* = 0.324	ρ = −0.158 *p* = 0.450	ρ = −0.133 *p* = 0.526	ρ = −0.189 *p* = 0.366	ρ = −0.003 *p* = 0.990	ρ = 0.086 *p* = 0.682
ICARS	ρ = −0.315 *p* = 0.117	ρ = −0.431 *p* = 0.028	ρ = −0.444 *p* = 0.023	ρ = −0.372 *p* = 0.061	ρ = −0.310 *p* = 0.123	ρ = −0.274 *p* = 0.175	ρ = −0.051 *p* = 0.803	ρ = −0.089 *p* = 0.667

CAU, Caudate nucleus; CER, cerebellum; HIP, hippocampus; L, left; R, right; PUT, putamen. *p* indicates uncorrected *p*-value. No correlations remained significant after FDR correction.

The brain-behavior relationships within the patient and healthy control groups are shown in Table 6. Stroop interference was negatively correlated with right hippocampal volume in patients (ρ = −0.538, *p* = 0.005, *p*_*FDR*_ = 0.040) (Figure 4A), whereas no such correlation was observed in healthy controls. Regarding associative learning performance, in healthy controls, fewer acquisition errors were correlated with larger volumes of both the left and right cerebellar cortices (ρ = −0.501, *p* = 0.008, *p*_*FDR*_ = 0.032; ρ = −0.520, *p* = 0.005, *p*_*FDR*_ = 0.032) (Figure 4D). In patients, learning performance was unrelated to cerebellar volumes. The number of trials to criterion was negatively correlated with right hippocampal volume (ρ = −0.576, *p* = 0.002, *p*_*FDR*_ = 0.016) (Figure 4B), whereas acquisition error rate was negatively correlated with right hippocampal volume (ρ = −0.662, *p* = 0.0002, *p*_FDR_ = 0.002), left hippocampal volume (ρ = −0.463, *p* = 0.017, *p*_FDR_ = 0.045), and right putamen volume (ρ = −0.519, *p* = 0.006, *p*_FDR_ = 0.026) (Figure 4C).

**TABLE 6:** Partial Spearman rank correlations (ρ) between behavioral performance and subcortical and cerebellar volumes controlling for eTIV.

Variable	L CAU	R CAU	L PUT	R PUT	L HIP	R HIP	L CER	R CER
HC								
Stroop interference time	ρ = −0.103	ρ = −0.211	ρ = −0.159	ρ = −0.218	ρ = −0.157	ρ = −0.183	ρ = 0.360	ρ = −0.371
	*p* = 0.608	*p* = 0.289	*p* = 0.429	*p* = 0.275	*p* = 0.434	*p* = 0.360	*p* = 0.065	*p* = 0.056
	*p*_FDR_ = 0.608	*p*_FDR_ = 0.496	*p*_FDR_ = 0.496	*p*_FDR_ = 0.496	*p*_FDR_ = 0.496	*p*_FDR_ = 0.496	*p*_FDR_ = 0.260	*p*_FDR_ = 0.260
CDTT acquisition-number of trials to criterion	ρ = −0.145	ρ = −0.144	ρ = −0.003	ρ = −0.107	ρ = −0.032	ρ = 0.159	ρ = −0.304	ρ = −0.344
	*p* = 0.469	*p* = 0.474	*p* = 0.989	*p* = 0.596	*p* = 0.873	*p* = 0.427	*p* = 0.123	*p* = 0.079
	*p*_FDR_ = 0.758	*p*_FDR_ = 0.758	*p*_FDR_ = 0.989	*p*_FDR_ = 0.795	*p*_FDR_ = 0.989	*p*_FDR_ = 0.758	*p*_FDR_ = 0.492	*p*_FDR_ = 0.492
CDTT acquisition-error rate (%)	ρ = −0.288	ρ = −0.380	ρ = −0.279	ρ = −0.338	ρ = −0.163	ρ = −0.063	**ρ = −0.501**	**ρ = −0.520**
	*p* = 0.145	*p* = 0.051	*p* = 0.158	*p* = 0.085	*p* = 0.416	*p* = 0.756	***p* = 0.008**	***p* = 0.005**
	*p*_FDR_ = 0.211	*p*_FDR_ = 0.136	*p*_FDR_ = 0.211	*p*_FDR_ = 0.170	*p*_FDR_ = 0.475	*p*_FDR_ = 0.756	*p*_FDR_ = 0.032*	*p*_FDR_ = 0.032*
CD								
Stroop interference time	ρ = −0.129	ρ = −0.047	ρ = −0.257	ρ = −0.266	ρ = −0.486	**ρ = −0.538**	ρ = 0.090	ρ = 0.048
	*p* = 0.538	*p* = 0.823	*p* = 0.215	*p* = 0.199	*p* = 0.014	***p* = 0.005**	*p* = 0.669	*p* = 0.820
	*p*_FDR_ = 0.823	*p*_FDR_ = 0.823	*p*_FDR_ = 0.430	*p*_FDR_ = 0.430	*p*_FDR_ = 0.056	*p*_FDR_ = 0.040*	*p*_FDR_ = 0.823	*p*_FDR_ = 0.823
CDTT acquisition-number of trials to criterion	ρ = −0.287	ρ = −0.238	ρ = −0.298	ρ = −0.285	ρ = −0.381	**ρ = −0.576**	ρ = 0.015	ρ = −0.007
	*p* = 0.155	*p* = 0.242	*p* = 0.139	*p* = 0.158	*p* = 0.055	***p* = 0.002**	*p* = 0.941	*p* = 0.971
	*p*_FDR_ = 0.253	*p*_FDR_ = 0.323	*p*_FDR_ = 0.253	*p*_FDR_ = 0.253	*p*_FDR_ = 0.220	*p*_FDR_ = 0.016*	*p*_FDR_ = 0.971	*p*_FDR_ = 0.971
CDTT acquisition-error rate (%)	ρ = −0.319	ρ = −0.304	ρ = −0.424	**ρ = −0.519**	**ρ = −0.463**	**ρ = −0.662**	ρ = 0.141	ρ = 0.081
	*p* = 0.112	*p* = 0.131	*p* = 0.031	***p* = 0.006**	***p* = 0.017**	***p* = 0.0002**	*p* = 0.490	*p* = 0.692
	*p*_FDR_ = 0.175	*p*_FDR_ = 0.175	*p*_FDR_ = 0.062	*p*_FDR_ = 0.026*	*p*_FDR_ = 0.045*	*p*_FDR_ = 0.002**	*p*_FDR_ = 0.560	*p*_FDR_ = 0.692

CAU, caudate nucleus; CD, patients with cerebellar degeneration; CER, cerebellum; HC, healthy controls; HIP, Hippocampus; L, left; R, right; PUT, putamen. *p* indicates uncorrected *p-*values; asterisks indicate FDR-corrected significance:

*p_FDR_ < 0.05,

**p_FDR_ < 0.01. FDR-corrected significant results are shown in bold.

**FIGURE 4:**
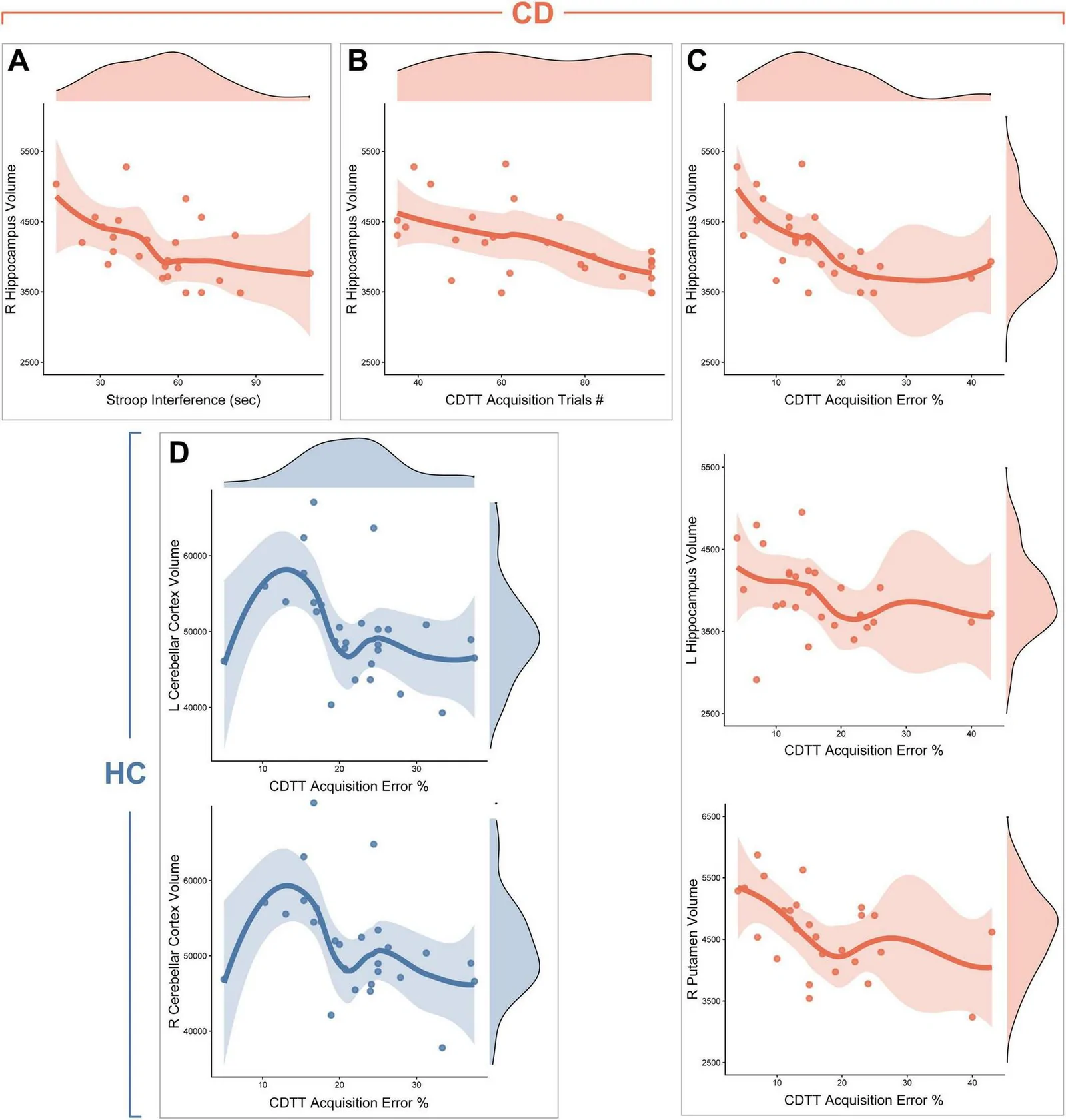
Scatter plots showing partial Spearman rank correlations between regional brain volumes and behavioral performance. **(A–C)** In patients, Stroop interference time (ρ = –0.538, *p* = 0.005, *p*_FDR_ = 0.040), the number of trials to criterion during the acquisition phase of the CDTT (ρ = –0.576, *p* = 0.002, *p*_FDR_ = 0.016) and CDTT error rate during the acquisition phase (ρ = –0.662, *p* = 0.0002, *p*_FDR_ = 0.002) were negatively correlated with right hippocampal volume. CDTT error rate was also negatively correlated with left hippocampal volume (ρ = –0.463, *p* = 0.017, *p*_FDR_ = 0.045) and right putamen volume (ρ = –0.519, *p* = 0.006, *p*_FDR_ = 0.026). **(D)** In healthy controls, CDTT error rate was negatively correlated with left cerebellar cortex volume (ρ = –0.501, *p* = 0.008, *p*_FDR_ = 0.032) and right cerebellar cortex volume (ρ = –0.520, *p* = 0.005, *p*_FDR_ = 0.032). All correlations were controlled for estimated total intracranial volume (eTIV). The solid line represents the LOESS-smoothed trend, and the shaded areas indicate the 95% confidence intervals. Marginal density plots illustrate the distribution of each variable. # denotes the number of trials. CD, patients with cerebellar degeneration; HC, healthy controls.

After controlling for Stroop interference time in addition to eTIV, right hippocampal volume remained negatively correlated with acquisition performance (ρ = −0.572, *p* = 0.003) and acquisition error rate (ρ = −0.677, *p* = 0.0003). Likewise, the correlation between right hippocampal volume and Stroop interference time remained significant after controlling for acquisition trials and error rate (ρ = −0.529, *p* = 0.009). These findings indicate that the observed correlations were not explained by shared variance between the two cognitive domains.

Follow-up ANCOVAs controlling for age, sex, and eTIV showed that the group × right hippocampal volume interaction was significant for the number of acquisition trials to criterion [*F*(1, 48) = 6.193, *p* = 0.016, η_p_^2^ = 0.114], indicating that the association between right hippocampal volume and acquisition speed differed between patients and healthy controls. For acquisition error rate, none of the group × regional brain volume interactions were significant: right putamen [*F*(1, 48) = 3.395, *p* = 0.072, *p*_FDR_ = 0.360, η_p_^2^ = 0.066], left hippocampus [*F*(1, 48) = 1.500, *p* = 0.227, *p*_FDR_ = 0.378, η_p_^2^ = 0.030], and right hippocampus [*F*(1, 48) = 1.667, *p* = 0.203, *p*_FDR_ = 0.378, η_p_^2^ = 0.034], left cerebellum [*F*(1, 48) = 0.535, *p* = 0.468, *p*_FDR_ = 0.468, η_p_^2^ = 0.011], and right cerebellum [*F*(1, 48) = 0.566, *p* = 0.456, *p*_FDR_ = 0.468, η_p_^2^ = 0.012].

## 4 Discussion

The present findings demonstrate impaired cognitive control and a selective impairment in the acquisition of feedback-based S-R associations in patients with cerebellar degeneration. Increased Stroop interference and errors indicate impaired cognitive control in patients, particularly involving inhibitory control and interference resolution. Likewise, slower acquisition together with increased learning errors suggests greater difficulty in the gradual formation of novel S-R associations. Among participants who reached the acquisition criterion, no significant between-group difference in transfer performance was observed. This pattern is compatible with greater difficulty in the acquisition of S-R associations than in their subsequent transfer to novel stimulus combinations. Together, these findings indicate that cerebellar degeneration primarily disrupts the gradual acquisition and refinement of novel S-R associations.

### 4.1 Impaired inhibitory control correlated with hippocampal volumes in cerebellar degeneration

The classic Stroop test requires the inhibition of a prepotent response tendency (i.e., word reading) and the selection of a task-relevant response (i.e., naming the ink color) under conditions of response conflict. Our patients with cerebellar degeneration showed poorer performance in the Stroop test, characterized by a greater number of errors and spontaneous corrections as well as longer interference times. This profile of impaired inhibitory control is consistent with the cerebellum’s established contribution to executive regulation through cerebello-cortical circuits (Schmahmann and Sherman, 1998; Stoodley and Schmahmann, 2018).

Interestingly, Stroop interference time was negatively correlated with right hippocampal volume in patients with cerebellar degeneration (ρ = –.538), with larger right hippocampal volume associated with shorter interference times, and this association remained significant following FDR correction. Notably, this association occurred in the absence of a significant between-group difference in hippocampal volume, suggesting that the relationship cannot simply be attributed to hippocampal atrophy in patients. Rather, although the present structural data cannot establish such a compensatory or functional mechanism, the observed negative correlation suggests that individual differences in the right hippocampal volume may be related to cognitive control in patients. Comparable brain-behavior relationships have been previously reported in Parkinson’s disease in the context of frontostriatal dysfunction. For example, Filoteo et al. (2014) demonstrated that poorer Stroop performance in nondemented Parkinson’s disease was associated not only with reduced right putamen volume but also with smaller right entorhinal cortex volume, whereas such brain-behavior relationships were absent in healthy controls. Likewise, although the hippocampus is unlikely to mediate inhibitory control directly, this association may be considered in light of proposed hippocampal contributions to memory-dependent aspects of cognitive control, such as maintenance of task context or relational representations.

### 4.2 Structural correlates of feedback-based S-R acquisition in healthy controls and cerebellar degeneration

Feedback-based acquisition of S-R associations has been attributed to frontostriatal mechanisms, whereas successful transfer has been linked to additional contributions from MTL systems (Myers et al., 2003; Shohamy et al., 2004). In the present study, acquisition error rate in healthy controls was not correlated with striatal volumes but was correlated with both left and right cerebellar cortex volumes, in line with the cerebellum’s proposed role in error-based learning across motor and non-motor domains (Seidler et al., 2013; Popa and Ebner, 2019).

The association between cerebellar volume and acquisition errors in healthy controls provides additional support for a role of the cerebellum in feedback-based learning, consistent with contemporary accounts emphasizing predictive processing and internal models (Ito, 2008). During feedback-based learning, the cerebellum has been proposed to contribute to predicting outcomes, detecting mismatches between expected and actual outcomes, and using prediction-error signals to guide subsequent behavior. Rather than serving as a storage system for learned associations, the cerebellum may facilitate efficient learning by accelerating the formation of predictive models linking environmental cues, responses, and outcomes (Wolpert et al., 1998; Miall and King, 2008; Popa and Ebner, 2019). Within this framework, cerebellar dysfunction may result in less efficient incremental learning, reflected in slower acquisition and increased acquisition errors. The absence of a significant group difference in transfer performance among participants who reached the criterion is compatible with the possibility that the primary difficulty lies in the gradual acquisition of associations rather than in the subsequent transfer of successfully acquired knowledge.

While previous findings suggest that the basal ganglia may contribute primarily to the incremental acquisition of S-R associations from response-contingent feedback, our findings extend this framework by suggesting that the cerebellum may also contribute to updating predictive representations based on feedback-related error signals. On the other hand, the absence of significant striatal volume-performance correlations in healthy controls may indicate that, within the range of normal structural variation, striatal volume does not strongly account for individual differences in acquisition performance.

The present findings are consistent with the acquisition–transfer pattern previously observed in patients with Parkinson’s disease, with impaired acquisition despite successful transfer of previously learned S-R associations. The structural findings demonstrated supratentorial involvement in addition to marked bilateral cerebellar atrophy in patients. The right caudate was the only supratentorial region showing significant volume reduction after correction for multiple comparisons, whereas the left caudate and left putamen exhibited smaller volumes that did not survive correction. These findings are consistent with evidence that cerebellar degeneration may involve extra-cerebellar structures within distributed cerebello-subcortical neural networks rather than the cerebellum in isolation.

The within-group brain-behavior correlations showed different patterns in patients and healthy controls; however, the exploratory interaction analysis indicated a significant group difference only in the association between right hippocampal volume and acquisition speed. In healthy controls, fewer acquisition errors were correlated with larger bilateral cerebellar volumes, consistent with a contribution of the cerebellum to feedback-based associative learning. In patients, however, higher acquisition error rate was associated with smaller right putamen and left and right hippocampal volumes, whereas slower acquisition was associated only with smaller right hippocampal volume. These within-group findings may be considered within the framework of complementary hippocampal-striatal learning systems. Early computational models proposed that the hippocampus and the corticostriatal system make distinct yet interacting contributions to learning, with the hippocampus supporting the formation of flexible stimulus representations and the striatum gradually acquiring S-R associations through feedback (Gluck and Myers, 1993). This framework was subsequently supported by experimental and neuroimaging studies demonstrating complementary and, under some conditions, competitive interactions between hippocampal and striatal memory systems (Packard and McGaugh, 1996; Knowlton et al., 1996; Packard and Knowlton, 2002; Poldrack and Packard, 2003; Yin and Knowlton, 2006). Within this context, the association between smaller right putamen volume and higher acquisition error rate in patients is consistent with the established role of the dorsal striatum (i.e., caudate and putamen) in the acquisition of S-R associations. At the same time, the significant interaction between group and right hippocampal volume for trials to criterion demonstrates that the relationship between right hippocampal volume and acquisition speed differed between patients and healthy controls, although the present structural data cannot establish whether this difference reflects altered functional recruitment or compensation.

Importantly, the regional volume reductions observed in patients did not directly correspond to the structures whose volumes were associated with acquisition performance. Although patients showed significant right caudate volume reduction, with trends toward smaller left caudate and left putamen volumes, acquisition performance was associated with right putamen and left and right hippocampal volumes, neither of which showed significant between-group volume reductions. Thus, the impaired acquisition of S-R associations cannot readily be explained simply by volume loss in the structures showing the significant atrophy. The within-group correlations therefore suggest that relatively preserved extra-cerebellar structures may be related to individual differences in cognitive performance, although a significant between-group difference in these brain-behavior relationships was demonstrated only for the association between right hippocampal volume and acquisition speed. This finding does not necessarily imply a primary role of the hippocampus in feedback-based learning. Rather, it may reflect the observed group difference in the relationship between right hippocampal volume and acquisition speed in the context of cerebellar degeneration. This interpretation is compatible with the multiple memory systems framework, which proposes that successful learning emerges through interactions among partially overlapping neural systems rather than isolated memory modules (Gluck and Myers, 1993; Poldrack and Packard, 2003).

Studies suggest that disruption of striatal learning mechanisms may increase the contribution of MTL systems that support relational and declarative representations, reflecting a shift in the balance between complementary memory systems rather than the recruitment of an entirely separate learning mechanism (Gluck and Myers, 1993; Packard and McGaugh, 1996; Poldrack and Packard, 2003). Consistent with this framework, Foerde and Shohamy (2011) showed that patients with Parkinson’s disease were impaired in learning from immediate feedback, whereas their learning performance was preserved when feedback was delayed by several seconds. Using task-based functional MRI, they further demonstrated that, in healthy individuals, immediate feedback engaged the striatum, whereas delayed feedback was associated with greater hippocampal activation. These findings suggest that the temporal availability of feedback influences the neural systems supporting learning, with immediate feedback preferentially engaging striatal mechanisms and delayed feedback shifting learning-related processing toward the hippocampus. Neuroimaging studies in Parkinson’s disease have similarly demonstrated increased recruitment of MTL regions during feedback-based category learning (Moody et al., 2004). These findings are further supported by evidence that Parkinson’s disease is accompanied by dynamic reorganization of learning-related neural networks as corticostriatal dysfunction progresses (Carbon et al., 2010). Moustafa et al. (2010) proposed a neurocomputational model of hippocampal-striatal interactions in associative learning, suggesting that feedback-based S-R learning is primarily mediated by the striatum under normal conditions. In their Parkinson’s disease model, striatal dysfunction resulting from dopamine depletion was simulated by reducing striatal learning efficiency, leading to slower acquisition of S-R associations. The model further suggests that, when striatal learning is impaired, the hippocampus may contribute more strongly to learning by encoding associative representations, thereby partially compensating for striatal deficits. Although this compensatory mechanism preserves learning performance to some extent, acquisition remains less efficient than when the striatal system is intact. Importantly, structural MRI cannot establish functional compensation or causal network reorganization. Nevertheless, the significant interaction between group and right hippocampal volume for acquisition speed demonstrates that this specific structural brain-behavior relationship differed between patients and healthy controls. This finding is compatible with a multiple-memory systems interpretation.

A lack of significant correlations with clinical variables suggests that the observed cognitive deficits and supratentorial structural involvement cannot readily be explained by disease duration or motor symptom severity. Instead, individual differences in cognitive performance were associated with hippocampal and putamen volumes, whereas no significant associations were observed with the clinical variables. Notably, the correlations between Stroop performance and regional hippocampal volumes remained significant after controlling for CDTT performance and eTIV, and correlations between CDTT acquisition performance and regional hippocampal and putamen volumes remained significant after controlling for Stroop interference time and eTIV. These findings indicate that the observed brain-behavior relationships were not attributable to shared variance between the two cognitive domains.

This study has several limitations that should be considered. First, the hereditary cerebellar ataxia cohort was genetically heterogeneous, comprising patients with different genetic subtypes, including both autosomal dominant and autosomal recessive forms. Although this reflects the clinical heterogeneity of hereditary cerebellar ataxias, subtype-specific differences in patterns of neurodegeneration and cognitive impairment may have contributed to variability in the observed brain-behavior relationships. Second, the neuroimaging analyses were restricted to a predefined set of hypothesis-driven regions of interest selected based on previous literature. Therefore, contributions of other cortical and subcortical regions to the observed cognitive deficits cannot be excluded. Third, transfer performance was examined only in participants who reached the acquisition criterion, resulting in the exclusion of a larger proportion of patients (8/27, 29.6%) than healthy controls (2/28, 7.1%). Therefore, the absence of a significant group difference in transfer performance applies only to this selected subgroup and should not be interpreted as evidence of preserved transfer ability in the overall patient group. Future studies with larger samples permitting subtype-specific analyses and whole-brain neuroimaging approaches are needed to determine whether the present findings generalize across hereditary cerebellar ataxia subtypes and extend beyond the predefined regions of interest.

Taken together, these findings indicate that cerebellar degeneration is associated with impaired cognitive control and slower acquisition of feedback-based S-R associations. The within-group correlation analyses revealed different patterns, with acquisition error rate correlated with bilateral cerebellar cortex volumes in healthy controls and with right putamen and left and right hippocampal volumes in patients. Importantly, the exploratory interaction analyses indicated a between-group difference specifically in the association between right hippocampal volume and acquisition speed. Future longitudinal studies combining structural MRI and task-based functional MRI will be important for determining whether this structural difference is accompanied by altered functional engagement of cerebellar, striatal, and hippocampal systems over the course of disease.

## 5 Conclusion

This study demonstrates that cerebellar degeneration impairs inhibitory control and incremental feedback-based S-R acquisition, while no significant difference in transfer performance was observed among participants who successfully acquired the associations. These findings indicate that the cognitive consequences of cerebellar disease are best understood within distributed learning systems rather than as isolated cerebellar dysfunction. Whereas acquisition error rate in healthy individuals was associated with bilateral cerebellar cortex volume, acquisition performance in patients was associated with right putamen and left and right hippocampal volumes, in the context of significant right caudate volume reduction. Importantly, direct interaction analyses demonstrated that the association between right hippocampal volume and acquisition speed differed between patients and healthy controls. This finding provides direct evidence of a group difference in the structural correlates of feedback-based acquisition, although structural MRI cannot determine whether this difference reflects altered functional recruitment, compensation, or network reorganization. Together, the findings support a distributed systems account of cognitive dysfunction in cerebellar degeneration, highlighting the contributions of cerebellar, striatal, and hippocampal structures to feedback-based learning. They further emphasize the importance of considering extra-cerebellar structures when investigating the neural basis of cognitive dysfunction in cerebellar degeneration.

## Data Availability

The raw data supporting the conclusions of this article will be made available by the authors, without undue reservation.

## Footnotes

1
https://surfer.nmr.mgh.harvard.edu
